# Unlocking the Potential of AI and Big Data in Cancer Research: Advances and Applications

**DOI:** 10.3390/cancers18081234

**Published:** 2026-04-14

**Authors:** Federico Mastroleo, Giulia Marvaso

**Affiliations:** 1 Division of Radiation Oncology, IEO European Institute of Oncology IRCCS, 20141 Milan, Italy; 2 Department of Oncology and Hemato-Oncology, University of Milan, 20122 Milan, Italy

Artificial intelligence (AI) and big data analytics have fundamentally reshaped the landscape of modern oncology. Over the past decade, the exponential growth of digitized clinical data (genomic sequencing, radiomics, digital pathology, electronic health records [EHRs]) has created unprecedented opportunities to apply machine learning (ML) and deep learning (DL) algorithms to problems that were previously intractable through conventional statistical approaches [1,2]. In radiation oncology, AI-driven autosegmentation of organs at risk and target volumes has emerged as one of the most mature clinical applications, with the potential to reduce inter-observer variability and planning time substantially [3]. Radiomics, the high-throughput extraction of quantitative imaging features, has gained traction as a non-invasive complement to tissue-based biomarkers, supporting outcome prediction models across multiple tumor sites [4]. In parallel, natural language processing (NLP) and large language models (LLMs) are beginning to unlock the vast unstructured textual information embedded in clinical notes, pathology reports, and the published literature, offering new avenues for automated data curation and clinical decision support [5,6].

## 1. Knowledge Gaps and How This Special Issue Addresses Them

Despite the rapid pace of methodological innovation, several critical knowledge gaps persist. First, the translational gap between proof-of-concept AI studies and clinically validated, prospectively tested tools remains substantial. Most published models have been developed and evaluated on retrospective, single-institution cohorts, with limited external validation and insufficient attention to generalizability across patient populations, imaging protocols, and institutional workflows [7].

Second, the interpretability and explainability of AI models continue to pose challenges for clinical adoption. While performance metrics such as area under the curve (AUC) are commonly reported, the mechanisms by which models arrive at their predictions are often opaque, limiting clinician trust and regulatory acceptance [8]. The recently published TRIPOD+AI statement [9] and the TRIPOD-LLM reporting guideline [10] represent important steps toward standardized, transparent reporting of prediction model studies, but their adoption in oncological research remains inconsistent.

Third, integrating AI tools into existing clinical information systems, including EHRs and treatment planning systems, presents substantial informatics and interoperability challenges. Real-time deployment of AI models requires not only technical infrastructure (e.g., FHIR-based interfaces, cloud or edge computing) but also robust governance frameworks to ensure data quality, patient privacy, and algorithmic fairness [11].

The contributions assembled in this Special Issue collectively address several of these gaps. The published articles span a wide thematic range, including deep learning for automated segmentation in radiotherapy planning, radiomics-based outcome prediction, bibliometric analyses of rapidly evolving oncological subfields, multi-omics data integration, and the application of AI to specific diagnostic and prognostic tasks. By bringing together original research and comprehensive reviews, this collection provides both methodological advances and critical evaluations of the state of the art, offering readers a cross-sectional view of how AI and big data are being translated into oncological practice.

## 2. Future Research Directions

Looking forward, several priority areas warrant focused investigation.

### 2.1. Prospective Validation and Clinical Implementation

The field must transition from retrospective model development to prospective, multi-institutional validation studies embedded within clinical trials or real-world deployment frameworks. Pragmatic trial designs, including stepped-wedge, cluster-randomized, and adaptive platform trials, are well-suited to evaluating AI-augmented workflows in oncology. Regulatory science for AI-based medical devices is evolving rapidly, and collaborative engagement between researchers, clinicians, and regulatory agencies will be essential [12,13].

### 2.2. Foundation Models and Large Language Models

The emergence of multimodal foundation models, pretrained on large-scale imaging, genomic, and textual corpora, represents a paradigm shift in how AI systems can be adapted to oncological tasks [14]. Transfer learning, few-shot learning, and prompt engineering may enable the development of high-performing models even in data-scarce clinical scenarios, such as rare tumor types or pediatric malignancies. The integration of LLMs with retrieval-augmented generation (RAG) architectures is particularly promising for clinical decision support systems that draw on institutional knowledge bases and current evidence.

### 2.3. Multi-Omics Integration and Digital Twins

The fusion of genomic, transcriptomic, proteomic, radiomic, and clinical data into unified predictive frameworks is an area of active development [15]. Longitudinal, multi-omics datasets that capture the evolution of disease biology and treatment response over time will be critical for building dynamic, patient-specific models, so-called digital twins, capable of informing adaptive treatment strategies. In silico clinical trials leveraging synthetic cohorts derived from such models may accelerate the evaluation of novel therapeutic approaches.

### 2.4. Federated Learning and Data Governance

Overcoming the barriers to multi-institutional data sharing without compromising patient privacy is a prerequisite for building generalizable AI models in oncology. Federated learning, differential privacy, and synthetic data generation are emerging as viable technical solutions [16], but their implementation at scale requires coordinated governance frameworks, standardized data models (e.g., OMOP-CDM, FHIR), and institutional commitment to interoperability.

### 2.5. Equity, Bias, and Ethical AI

As AI tools become increasingly embedded in clinical decision-making, ensuring that they perform equitably across diverse patient populations is paramount. Algorithmic auditing, fairness-aware model training, and the inclusion of underrepresented populations in training datasets are critical safeguards against perpetuating or amplifying existing health disparities [17].

## 3. Conclusions

This Special Issue illustrates both the breadth and the depth of current research at the intersection of AI, big data, and cancer. The contributions highlight meaningful progress in autosegmentation, outcome modeling, bibliometric characterization of emerging fields, and the integration of multi-dimensional data for clinical decision support. At the same time, they underscore the work that remains: prospective validation, clinical implementation, regulatory alignment, and equitable deployment.

We are grateful to all the authors who contributed their work to this Special Issue, to the reviewers who ensured the rigor of the published articles, and to the editorial team at *Cancers* for their support throughout the process. We hope that this collection will serve as both a reference for the current state of the field and a stimulus for the next generation of studies that bring AI-driven cancer research closer to clinical impact.
